# Association between periodontal disease and chronic obstructive pulmonary disease: an umbrella review

**DOI:** 10.3389/froh.2026.1728405

**Published:** 2026-03-27

**Authors:** Fredy Hugo Cruzado-Oliva, Silvia Elizabet Reyes-Narváez, Edi William Aguilar-Urbina, Edward Demer Infantes-Ruiz, Heber Isac Arbildo-Vega, Rubén Aguirre-Ipenza, Franz Tito Coronel-Zubiate

**Affiliations:** 1 Faculty of Stomatology, Stomatology School, Universidad Nacional de Trujillo, Trujillo, Peru; 2 Faculty of Medical Sciences, School of Nursing, Universidad Nacional Santiago Antunez de Mayolo, Ancash, Peru; 3 Faculty of Medicine, Medicine School, Universidad Nacional de Trujillo, Trujillo, Peru; 4 Faculty of Health Science, Stomatology School, Universidad César Vallejo, Piura, Peru; 5 Faculty of Dentistry, Dentistry School, Universidad San Martín de Porres, Chiclayo, Peru; 6 Faculty of Health Sciences, Universidad Continental, Lima, Peru; 7 Faculty of Health Sciences, Stomatology School, Universidad Nacional Toribio Rodríguez de Mendoza de Amazonas, Chachapoyas, Peru

**Keywords:** chronic obstructive pulmonary disease, oral-systemic health, periodontal disease, systematic review, umbrella review

## Abstract

**Aim:**

To evaluate the strength and methodological quality of evidence regarding the association between periodontal disease (PD) and chronic obstructive pulmonary disease (COPD) through an umbrella review.

**Materials and methods:**

A comprehensive search was conducted through April 2025 in PubMed, Cochrane Library, Scopus, Embase, Web of Science, Google Scholar, ProQuest Dissertations and Theses, and OpenGrey. Systematic reviews (SRs), with or without meta-analysis, investigating the association between PD and COPD were included without time or language restrictions. Narrative reviews, primary studies, protocols, and non-systematic reports were excluded. Methodological quality was assessed using AMSTAR-2. The degree of primary study overlap was calculated using the Corrected Covered Area (CCA) index. Due to high overlap (CCA = 14.45%) and clinical heterogeneity, no *de novo* meta-analysis was performed, and a structured qualitative synthesis was conducted.

**Results:**

Of 313 identified records, 12 SRs comprising 145 primary studies were included. Six reviews were rated as high confidence, one as low confidence, and five as critically low confidence according to AMSTAR-2. Most SRs reported a positive association between PD and COPD. However, the magnitude and statistical significance of the association varied according to periodontal parameters, COPD outcomes, and smoking status.

**Conclusion:**

Current evidence suggests a likely association between PD and COPD, although the relationship may be influenced by shared risk factors such as smoking and methodological heterogeneity. Integrating periodontal assessment into COPD management may be clinically relevant, but high-quality prospective studies are needed to clarify causal pathways.

**Systematic Review Registration:**

Open Science Framework (OSF), https://doi.org/10.17605/OSF.IO/YTXB6.

## 1 Introduction

Periodontal disease (PD) is a chronic inflammatory condition affecting the supporting tissues of the teeth and is widely prevalent worldwide (1–3). This high prevalence establishes PD as one of the most common oral health challenges and a significant contributor to systemic morbidity. PD is associated with numerous systemic conditions, including diabetes and cardiovascular diseases (2, 4–10), underscoring its clinical and epidemiological relevance. Consequently, the scientific community has explored the potential link between PD and chronic respiratory diseases, particularly chronic obstructive pulmonary disease (COPD) (2, 4).

COPD is a common respiratory disorder characterized by persistent bronchial obstruction and represents a major global public health concern. Its estimated prevalence is approximately 10%–13% in the general adult population (11) and 12.6% in individuals over 40 years of age (12). As the third leading cause of death worldwide, COPD is responsible for more than 3 million deaths annually (2, 4, 13, 14). The disease is characterized by symptoms such as dyspnea, chronic cough, and sputum production, with severity ranging from moderate to severe (4, 11, 15, 16). Due to its high burden and substantial healthcare costs (1, 4, 17), several modifiable risk factors have been identified, including air pollution and occupational exposures; however, PD and COPD share a critical common risk factor: smoking (1, 4, 11, 13).

Both conditions appear to be linked by a common pathophysiological axis: a chronic inflammatory response characterized by neutrophil activation and the systemic release of proinflammatory cytokines, such as IL-1, IL-6, and TNF-α (4, 13).

From a mechanistic perspective, it is postulated that periodontal pockets serve as reservoirs for pathogens and inflammatory mediators. Through microaspiration, these elements reach the lower respiratory tract, exacerbating bronchial inflammation and accelerating the decline of forced expiratory volume in one second (FEV_1) (2, 11, 18–22).

Observational studies suggest that periodontal therapy may reduce the frequency of exacerbations and improve long-term survival (11, 23–32).

Nevertheless, the interpretation of this relationship is not without controversy. Despite the robust epidemiological association reported, previous systematic reviews (SRs) have indicated that the effect of PD on COPD loses statistical significance after rigorous adjustment for smoking intensity (1). This inconsistency suggests that the association may be mediated by unresolved confounding variables, creating uncertainty regarding causality and the clinical utility of dental interventions in respiratory patients.

Despite the increasing number of systematic reviews (SRs) on this topic, findings remain inconsistent due to methodological heterogeneity, varying definitions of periodontal parameters, and the inclusion of overlapping primary studies. To address these challenges, this umbrella review was conducted to provide a high-level synthesis of the available evidence and to critically evaluate the methodological quality of the included SRs using the AMSTAR-2 tool. Additionally, we assessed the degree of primary study overlap using the Corrected Covered Area (CCA) metric, an essential component for evaluating evidence redundancy and the independence of findings.

This review adds value to the existing literature by offering an integrative perspective that transcends individual SRs. It summarizes the strength and consistency of the association between PD and COPD, highlights research gaps, and provides guidance for future studies. Therefore, the objective of this umbrella review is to evaluate the robustness and quality of the evidence supporting the association between PD and COPD in adults, based on existing systematic reviews.

The primary research question was: “What is the current strength and methodological quality of the evidence from systematic reviews regarding the association between periodontal disease and chronic obstructive pulmonary disease in adults?” This review aims not only to summarize the findings of existing systematic reviews but also to evaluate their methodological rigor and the consistency of their conclusions.

## 2 Materials and methods

### 2.1 Protocol and registration

A protocol was developed in accordance with the Preferred Reporting Items for Systematic Review and Meta-Analysis Protocols (PRISMA-P) guidelines (33) and was registered in the Open Science Framework (OSF) repository under the DOI code 10.17605/OSF.IO/YTXB6. This review adheres to the reporting standards outlined in the Preferred Reporting Items for Overviews of Systematic Reviews including Harms (PRIO-harms) checklist (34). Ethical approval was not deemed necessary for this umbrella review.

The research question was formulated using the PECO framework (Population, Exposure, Comparison, Outcomes) as follows:

- Population: Adults (≥18 years old) with or without COPD.
- Exposure: Periodontal disease (including gingivitis and periodontitis).
- Comparison: Periodontal health (absence of periodontal disease).
- Outcome: Presence, severity, or exacerbations of COPD.

### 2.2 Eligibility criteria and results of interest

Eligible studies included systematic reviews (SRs), with or without meta-analyses, without restrictions on publication date or language, that investigated primary studies exploring the association between PD and COPD. Excluded were literature or narrative reviews, rapid reviews, interventional studies, observational studies, preclinical and basic research, abstracts, commentaries, case reports, protocols, personal opinions, letters, and posters.

Included systematic reviews were grouped for synthesis according to the respiratory outcomes evaluated (COPD diagnosis, severity, exacerbations) and periodontal parameters assessed, allowing structured comparison across outcome domains.

### 2.3 Sources of information, search strategy and additional search for primary studies

On April 4, 2025, an electronic search was conducted in five databases: PubMed, Cochrane Database, Embase, Web of Science, and Scopus. Gray literature was explored via Google Scholar, ProQuest Dissertations and Theses, and OpenGrey. Additionally, reference lists of the included studies were manually screened. Retrieved articles were managed using reference management software (Zotero® 6.0, Center for History and New Media, Fairfax, Virginia, USA), and duplicate entries were removed. The complete electronic search strategies, including Boolean operators, Medical Subject Headings (MeSH) terms, free-text keywords, and applied filters, are provided in Supplementary Material 1 to ensure full reproducibility.

### 2.4 Data management and selection process

Identified articles were uploaded to Rayyan®, an online platform managed by the Qatar Computing Research Institute (Doha, Qatar). The study selection was conducted in two phases: initially, two reviewers EA and SR independently screened titles and abstracts. In phase two, the same reviewers independently assessed full-text articles. Discrepancies were resolved by consultation with a third reviewer FCO.

### 2.5 Data collection process

Data were independently and in duplicate extracted by two reviewers EI and FCZ using a pre-designed data extraction table. The data were then cross-verified, and any discrepancies were resolved by consulting a third author FCO. Extracted data included: authors, year of publication, study design, overall review design, number of studies included in qualitative and quantitative syntheses, outcomes, main conclusions, and any mention of frameworks or methodologies such as PRISMA, PROSPERO, GRADE (Grading of Recommendations Assessment, Development and Evaluation), and meta-analysis. No attempts were made to contact original study authors for missing or unclear data, as only published summary data from the included systematic reviews were extracted. All outcomes compatible with the predefined domains (COPD diagnosis, severity, exacerbations, and periodontal parameters) were extracted as reported by the original systematic reviews, without restriction by time point or analytical model. When information was unclear or incompletely reported in the original systematic reviews, data were recorded as reported without imputation or additional assumptions.

### 2.6 Methodological quality assessment, certainty of evidence and risk of bias

Two reviewers (HA and RI) independently and in duplicate assessed the methodological quality of the included SRs using the AMSTAR-2 (A Measurement Tool to Assess Systematic Reviews) checklist (35), achieving a Kappa calibration score of 0.85. AMSTAR-2 evaluates the methodological quality of systematic reviews across 16 domains, each rated as “yes,” “no,” or “partially yes.” Overall confidence in each review was classified as high, moderate, low, or critically low, following the criteria proposed by Shea et al. (35).

The overlap of primary studies across reviews was quantified using the Corrected Covered Area (CCA) index, as proposed by Pieper et al. (36). A citation matrix was constructed (see Supplementary Material 5) to determine the frequency with which primary studies were represented across the included reviews.

Reporting bias at the umbrella review level was not formally assessed, as no *de novo* quantitative meta-analysis was conducted. Certainty of evidence was also not reassessed at the umbrella level. However, when available, GRADE assessments reported by the original systematic reviews were extracted and documented.

### 2.7 Summary of measures

For SRs without meta-analysis, the summarized findings of the included primary studies were considered. When meta-analyses were available, results were extracted based on the reported effect measures, including odds ratios (OR), mean differences (MD), and weighted mean differences (WMD), to evaluate the association between PD and COPD.

A qualitative synthesis was prioritized due to the high heterogeneity in clinical parameters and reporting styles across the included SRs. Although a structured comparison of effect sizes (OR, MD, WMD) is provided as reported by the original authors, no formal *de novo* meta-analysis was conducted to avoid “double-counting” primary studies and to preserve methodological integrity, given the high degree of overlap identified (CCA = 14.45%).

Systematic reviews were considered eligible for each outcome-specific synthesis if they reported quantitative estimates or narrative conclusions corresponding to the predefined outcome domains. No statistical conversions or recalculations were undertaken; effect measures were extracted and presented exactly as originally reported.

Findings were summarized in a structured narrative format and in detailed tables (Supplementary Material 7), organized according to outcome categories. No additional subgroup analyzes or meta-regressions were conducted at the umbrella level, given the decision not to perform a *de novo* meta-analysis. Similarly, no sensitivity analyzes were undertaken for the same methodological reasons.

### 2.8 Summary of results

The main results from the included SRs were summarized and categorized according to respiratory-related outcomes. These categories included COPD diagnosis, COPD severity and exacerbations, smoking, and various periodontal indicators (e.g., number of remaining teeth, oral hygiene index, gingival index, plaque index, probing depth, bleeding on probing, clinical attachment level, and alveolar bone loss).

## 3 Results

### 3.1 Study selection

**Figure 1:**
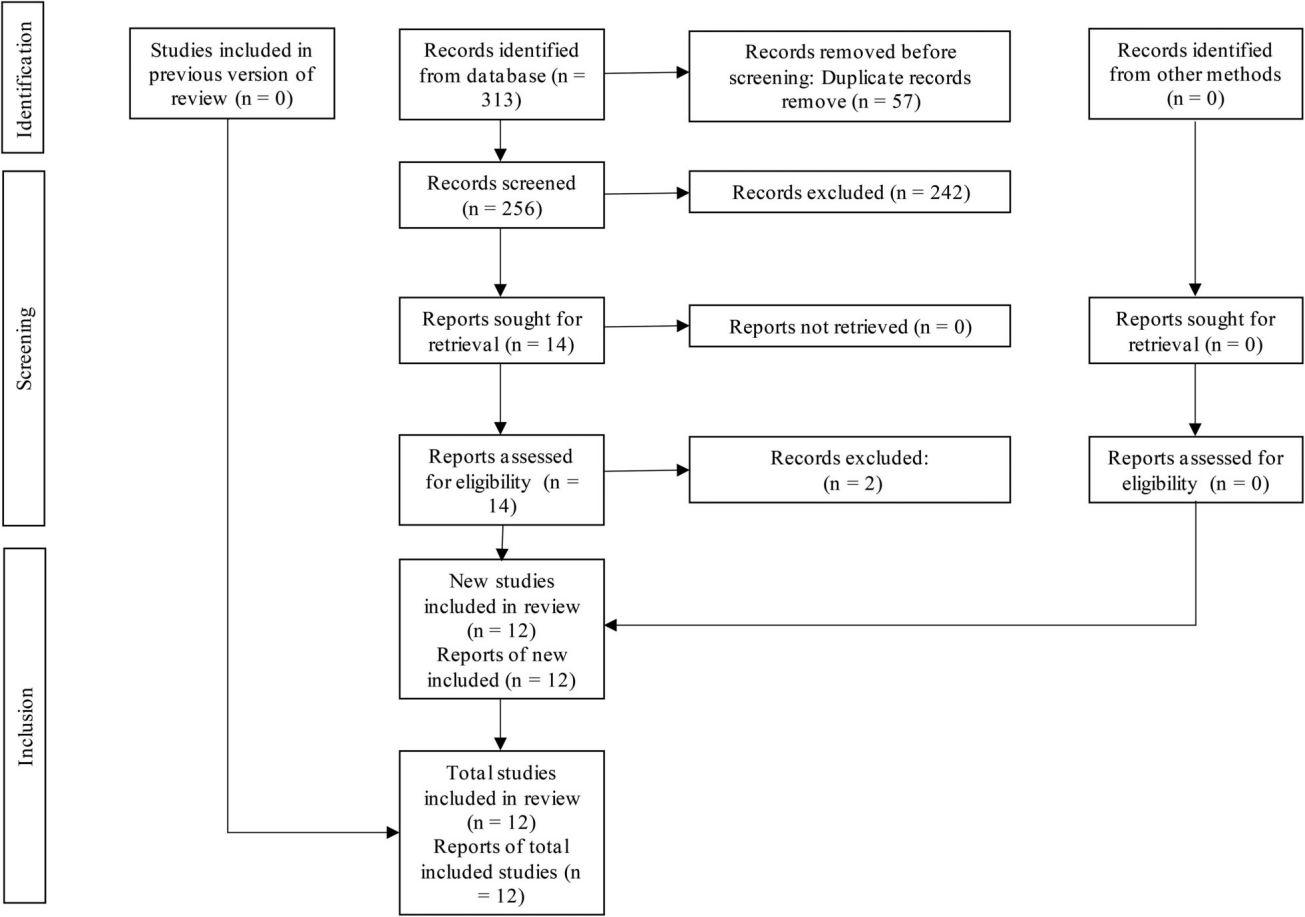
PRISMA flow diagram showing the selection process of studies included in the systematic review, from initial identification to final inclusion.

The initial database search identified 313 records, of which 256 remained after duplicate removal. Following title and abstract screening, 14 articles were considered eligible for full-text assessment. Ultimately, 12 systematic reviews met the inclusion criteria and were included in the qualitative synthesis. Reasons for exclusion at the full-text stage are detailed in Supplementary Material 2. The characteristics of the included studies are presented in Supplementary Material 3. The full process of study identification and selection is illustrated in Figure 1.

The populations included in the systematic reviews were predominantly adults aged 40 years or older, reflecting the typical demographic affected by COPD. However, variability was observed in how periodontal disease was defined; while some reviews utilized the CDC/AAP criteria, others relied on individual clinical parameters such as Probing Depth (PD) ≥ 3 mm or Clinical Attachment Loss (CAL). Outcome parameters were largely consistent regarding clinical indicators (PI, GI, CAL, PD), but heterogeneity existed in the reporting of COPD severity (GOLD stages vs. frequency of exacerbations).

### 3.2 Assessment of methodological quality and quality of evidence

Six SRs were considered high confidence (1, 27, 28, 37–39), one SR was low confidence (40) and five SRs were critically low confidence (41–45). Detailed assessments are provided in Supplementary Material 4. GRADE assessments were reported in only one review (28), as detailed in Supplementary Material 5.

### 3.3 Overlapping

One hundred and forty-five primary studies were identified in the SRs. The degree of overlap according to the CCA index is 14.45%, and this value indicates “high overlap”. Seven studies were duplicated in two reviews, while seven appeared in three. Additionally, five studies were found in four reviews, and five overlapped in five. Similarly, one study was included in six reviews, one in seven, two in eight, and one in nine. Further details on the overlap and characteristics of the primary studies can be found in Supplementary Material 6.

Given the high degree of primary study overlap (CCA = 14.45%) and heterogeneity across the included systematic reviews, no *de novo* meta-analysis was performed in this umbrella review. Instead, we provide a structured narrative synthesis of reported effect sizes, as detailed below.

### 3.4 Synthesis of results

A structured synthesis of the findings across included SRs is presented below, organized according to the main clinical parameters evaluated in the context of COPD. Details of each SR's findings, including pooled estimates and significance levels, are summarized in Supplementary Material 7.

### 3.5 Chronic obstructive pulmonary disease (COPD)

Nine included SRs (1, 28, 37, 39–41, 43–45) reported an association between PD and COPD. Five of these (1, 28, 37, 39, 43) performed meta-analyses and found odds ratios (ORs) ranging from 1.20 (CI: 1.09–1.32) (1) to 2.08 (CI: 1.48–2.91) (43). Mushtaq et al. (41), Azarpazhooh et al. (40), Scannapieco et al. (44) and Garcia et al. (45) also reported a significant association between PD and COPD.

### 3.6 COPD exacerbations

One SR (38) found an association between PD and COPD exacerbations, while another (1) did not. The latter performed a meta-analysis yielding an OR of 1.18 (CI: 0.71–1.21). Kelly et al. (38) noted a possible positive correlation between improved periodontal health, reduced hospitalizations, and better quality of life in COPD patients.

### 3.7 Smoking

One included SR (1) reported no significant association between PD and smoking or smoking intensity in individuals with COPD. Meta-analysis from the study yielded an OR of 1.46 (CI: 0.92–2.31) for smokers, 0.93 (CI: 0.72–1.21) for non-smokers, and 1.14 (CI: 0.86–1.51) for smoking intensity.

### 3.8 Periodontal indicators

#### 3.8.1 Number of remaining teeth

Two SRs (27, 37) identified a reduction in the number of remaining teeth among individuals with COPD. The meta-analyses reported a weighted mean difference (WMD) of −3.51 (CI: −4.66 to −2.35) (37) and a mean difference (MD) of −3.73 (CI: −5.12 to −2.33) (27).

#### 3.8.2 Oral hygiene index

Both SRs also found higher oral hygiene index scores in COPD patients, with WMD and MD values of 0.81 (CI: 0.48–1.14) (37) and 0.80 (CI: 0.33–1.28) (27), respectively.

#### 3.8.3 Gingival index

Regarding the gingival index, the same reviews (27, 37) observed elevated values among COPD patients. The meta-analyses yielded a WMD of 0.41 (CI: 0.–0.70) (37) and an MD of 0.37 (CI: 0.04–0.69) (27).

#### 3.8.4 Plate index

An association between PD and plaque index was also consistently reported in these SRs. The pooled estimates showed a WMD of 0.29 (CI: 0.11–0.47) (37) and an MD of 0.23 (CI: 0.04–0.41) (27).

In addition, SR (42) detected this association exclusively in smokers, with reported ORs of 3.99 (CI: 2.58–6.16) for smokers, 2.18 (CI: 0.89–5.33) for former smokers, and 1.52 (CI: 0.76–3.05) for non-smokers.

#### 3.8.5 Probing depth

Two SRs (27, 37) also found increased probing depth in COPD patients, with WMD and MD values of 0.33 (CI: 0.11–0.55) and 0.26 (CI: 0.02–0.50), respectively.

Separately, SR (42) reported this association only in non-smokers, with ORs of 0.43 (CI: 0.14–1.31) for smokers and 0.30 (CI: 0.15–0.62) for non-smokers.

#### 3.8.6 Bleeding on probing and bleeding index

SR (27) reported a significant association between PD and bleeding on probing (MD: 6.88, CI: 5.49–8.27), but not when using the bleeding index (MD: 0.24, CI: −0.11–0.59).

Similarly, SR (42) found no significant association between PD and bleeding index in any smoking subgroup, with ORs of 1.35 (CI: 0.57–3.24) for smokers, 0.37 (CI: 0.13–1.01) for former smokers, and 1.19 (CI: 0.58–2.46) for non-smokers.

#### 3.8.7 Clinical attachment level (CAL)

An association between PD and greater CAL in COPD patients was reported in two SRs (27, 37), with a WMD of 0.69 (CI: 0.45–0.93) and an MD of 0.48 (CI: 0.28–0.68), respectively.

SR (42) also evaluated this parameter and found a marginal association in smokers (OR: 0.99, CI: 0.98–1.00), and non-significant values for former smokers and non-smokers.

#### 3.8.8 Alveolar bone loss

Two SRs (1, 37) observed a significant association between PD and alveolar bone loss in COPD patients (OR: 1.98, CI: 1.32–2.97; WMD: 0.63, CI: 0.26–0.99). In contrast, SR (27) reported only a modest difference (MD: 0.13, CI: 0.00–0.25).

Taken together, the evidence indicates a probable association between PD and several COPD-related outcomes, although the strength and consistency of the associations vary across periodontal parameters and SR quality levels. Smoking status appears to be a key effect modifier, and further studies are warranted to delineate causal pathways.

## 4 Discussion

This umbrella review, which included 12 systematic reviews (6 of high confidence), confirmed a significant association between periodontal disease (PD) and chronic obstructive pulmonary disease (COPD). Our findings indicate that PD is linked to both the prevalence and exacerbations of COPD. Conversely, no association was found between PD and smoking or its intensity in COPD patients, nor with the gingival bleeding index. Nevertheless, positive associations were observed between PD and several clinical periodontal parameters in COPD patients, including number of remaining teeth, oral hygiene index, plaque index, gingival index, probing depth, bleeding on probing, clinical attachment level, and alveolar bone loss.

### 4.1 Biological plausibility

One plausible pathophysiological explanation for the PD–COPD association lies in shared inflammatory and microbiological mechanisms. Chronic gingivitis and periodontitis promote systemic inflammation mediated by proinflammatory cytokines such as IL-6, IL-1β, and TNF-α, which can exacerbate the underlying pulmonary inflammatory response in COPD (24, 46).

Additionally, the aspiration or migration of periodontal pathogens (e.g., Porphyromonas gingivalis, Tannerella forsythia, Treponema denticola) to the respiratory tract may lead to bronchial colonization and chronic lung infections. These microorganisms may reach the lungs via contaminated saliva or nocturnal microaspiration, altering pulmonary microbiota and potentially advancing COPD progression. Their presence in the lungs may increase both local and systemic inflammation, forming a vicious cycle that biologically explains the observed PD–COPD link (24, 46).

Recent evidence from the oral-lung axis suggests that the migration of periodontal pathogens, such as Porphyromonas gingivalis, does not merely represent colonization but actively disrupts pulmonary immune homeostasis, as discussed by Gaeckle et al. (47), Feng et al. (48) and Xiong et al. (49). This supports our findings regarding the association between PD and increased COPD exacerbations.

### 4.2 Consistency of evidence and the role of confounders

Our findings are consistent with other studies, such as Molina et al. (28), who found a positive association between periodontitis and COPD in a meta-analysis. Similarly, Shi et al. (27), reported worse periodontal health in COPD patients compared to healthy individuals. Even Mendelian randomization studies suggest that periodontitis may be an independent risk factor for COPD (46), supporting the notion that PD not only correlates with COPD but may also influence its severity and progression.

However, the literature is not entirely consistent. Yang et al. (1), after thoroughly adjusting for smoking, concluded that the association between periodontitis and COPD or its exacerbations was no longer statistically significant. This suggests that smoking—a shared risk factor—may partly mediate the observed PD–COPD association. Thus, confounding factors such as smoking history and systemic comorbidities must be considered when interpreting these results.

The discrepancies observed between reviews, such as the lack of significance in some smoking-adjusted models vs. the strong associations in others, may stem from differences in the primary studies’ geographic locations, sample sizes, and the specific periodontal indices prioritized. For instance, reviews focusing on hospitalized patients reported stronger associations than those involving community-dwelling populations, suggesting that disease severity acts as a major source of heterogeneity.

Regarding COPD exacerbations, our results align with previous literature indicating an association with PD. Xiong et al. (13), reported a significantly higher frequency of exacerbations in patients with periodontitis and observed that periodontal interventions reduced such events.

Similarly, Apessos et al. (31), documented in their SR that periodontal treatment in COPD patients was associated with more favorable respiratory outcomes, including reduced hospitalization and mortality rates. Although randomized controlled trials on this topic are still limited, preliminary findings suggest that improving oral health may positively influence respiratory prognosis by reducing bacterial burden and systemic inflammation.

In contrast, no significant association was found between PD and the gingival bleeding index in COPD patients, consistent with Shi et al. (27), who reported no significant difference in this parameter between COPD and non-COPD individuals. While other indicators like probing depth or bleeding on probing were elevated in COPD patients, the gingival bleeding index did not differ significantly, suggesting that not all periodontal parameters are equally affected.

It is also noteworthy to mention that the overlap among the studies, as measured by the CCA index, was 14.45%. This redundancy may artificially inflate the consistency of the evidence, as repeated use of the same data can give a misleading impression of increased precision. Methodologically, such overlap highlights the need to coordinate efforts and avoid duplication in future reviews. Thus, while findings appear consistent, they should be interpreted cautiously given the potential for publication and redundancy bias.

### 4.3 Clinical and research implications

Clinically, these results underscore the importance of integrating periodontal evaluation into COPD management. Collaboration between pulmonologists and dental professionals could help address oral factors that may worsen respiratory disease. As suggested by Apessos et al., it is reasonable to recommend that COPD patients maintain good oral health. Strategies such as oral hygiene education, antiseptic mouthwashes, and periodontal treatment programs may be integrated into COPD care protocols to potentially reduce exacerbations and improve quality of life. Indeed, the evidence suggests that improving oral health is associated with better lung function and clinical outcomes, including reduced mortality and hospitalization (31).

Clinically, we recommend that pulmonologists include a basic oral health screening as part of the initial COPD assessment. Specifically, patients should be referred for professional periodontal cleaning at least twice a year. For patients with limited mobility or severe dyspnea, the use of 0.12% chlorhexidine rinses or high-concentration fluoride toothpastes may serve as adjunctive measures to reduce the oral bacterial load and prevent microaspiration-related exacerbations.

For future research, prospective cohort studies and randomized clinical trials are needed to assess the impact of periodontal treatment on COPD progression. As noted by Shi et al. (27), high-quality, well-designed studies are necessary to validate the influence of PD on pulmonary outcomes. Ideally, future research should carefully control for smoking and other comorbidities, standardize periodontitis definitions, and evaluate outcomes such as lung function, exacerbation frequency, hospitalization rates, and inflammatory markers. It is also essential to reduce research redundancy by conducting new meta-analyses that avoid data overlap. Specifically, there is a critical need for large-scale prospective studies involving non-smokers with COPD. Isolating this cohort would allow researchers to determine the strength of the oral-lung axis without the overwhelming confounding effect of tobacco use, potentially revealing unique inflammatory pathways shared between PD and COPD.

In summary, our findings reinforce the idea that PD and COPD are epidemiologically and potentially pathogenetically linked. Although current evidence supports this association (and a possible benefit of periodontal therapy), it should be interpreted with caution due to confounding factors (e.g., smoking) and methodological heterogeneity. Identifying and managing periodontal health in patients with COPD could be valuable adjunctive interventions, but further longitudinal studies and clinical trials are needed to conclusively establish the effect of periodontal treatment on respiratory outcomes.

## 5 Conclusions

In conclusion, the current body of high-confidence evidence suggests a significant association between periodontal disease (PD) and chronic obstructive pulmonary disease (COPD). However, this relationship appears to be partially mediated by shared risk factors—most notably smoking—and confounding remains a key limitation. The consistency of this association varies across systematic reviews, particularly those with lower methodological quality or without proper adjustment for founders. While periodontal screenings should be considered in respiratory healthcare settings, the lack of robust longitudinal interventional studies limits our ability to draw causal inferences. Future research should prioritize well-designed, smoking-adjusted prospective trials to clarify whether periodontal therapy can independently influence the course of COPD.

## Data Availability

The original contributions presented in the study are included in the article/Supplementary Material, further inquiries can be directed to the corresponding author/s.
