# Supplementary material for: Association between periodontal disease and chronic obstructive pulmonary disease: an umbrella review
Source: Front Oral Health. 2026 Mar 27;7:1728405. doi: 10.3389/froh.2026.1728405 (PMC13066220; doi:10.3389/froh.2026.1728405)
Supplement: Supplementary file 1 [file Table1.docx]

Supplementary Material 1. Search strategy for each search engine

| Database | Strategy | Number of studies |
| --- | --- | --- |
| Pubmed | ("pulmonary disease, chronic obstructive"[MeSH Terms] OR "chronic obstructive pulmonary"[Title/Abstract] OR "obstructive pulmonary disease*"[Title/Abstract] OR "COPD"[Title/Abstract] OR "COAD"[Title/Abstract] OR "obstructive airway disease*"[Title/Abstract] OR "chronic airflow obstruction*"[Title/Abstract] OR "lung diseases, obstructive"[MeSH Terms] OR "obstructive lung disease*"[Title/Abstract]) AND ("periodontal diseases"[MeSH Terms] OR "periodontal disease*"[Title/Abstract] OR "parodontos*"[Title/Abstract] OR "gingival diseases"[MeSH Terms] OR "gingival disease*"[Title/Abstract] OR "gingivos*"[Title/Abstract] OR "gingivitis"[MeSH Terms] OR "gingiviti*"[Title/Abstract] OR "periodontitis"[MeSH Terms] OR "periodontiti*"[Title/Abstract] OR "aggressive periodontitis"[MeSH Terms] OR "chronic periodontitis"[MeSH Terms] OR "chronic periodontiti*"[Title/Abstract] OR "periapical periodontitis"[MeSH Terms] OR "periodontal abscess"[MeSH Terms] OR "periodontal abscess*"[Title/Abstract] OR "periodontal pocket"[MeSH Terms] OR "periodontal pocket*"[Title/Abstract]) AND ("systematic review"[Publication Type] OR "systematic reviews as topic"[MeSH Terms] OR "systematic review"[All Fields] OR "meta analysis"[Publication Type] OR "meta analysis as topic"[MeSH Terms] OR "meta analysis"[All Fields]) | 26 |
| Cochrane Database | ([mh "pulmonary disease, chronic obstructive"] OR "chronic obstructive pulmonary":ti,ab OR ("obstructive pulmonary" NEXT disease*):ti,ab OR COPD:ti,ab OR COAD:ti,ab OR ("obstructive airway" NEXT disease*):ti,ab OR ("chronic airflow" NEXT obstruction*):ti,ab OR [mh "lung diseases, obstructive"] OR ("obstructive lung" NEXT disease*):ti,ab) AND ([mh "periodontal diseases"] OR ("periodontal" NEXT disease*):ti,ab OR parodontos*:ti,ab OR [mh "gingival diseases"] OR ("gingival" NEXT disease*):ti,ab OR gingivos*:ti,ab OR [mh gingivitis] OR gingiviti*:ti,ab OR [mh periodontitis] OR periodontiti*:ti,ab OR [mh "aggressive periodontitis"] OR [mh "chronic periodontitis"] OR ("chronic" NEXT periodontiti*):ti,ab OR [mh "periapical periodontitis"] OR [mh "periodontal abscess"] OR ("periodontal" NEXT abscess*):ti,ab OR [mh "periodontal pocket"] OR ("periodontal" NEXT pocket*):ti,ab) AND ("systematic review":pt OR [mh "systematic reviews as topic"] OR "systematic review" OR "meta analysis":pt OR [mh "meta analysis as topic"] OR "meta analysis") | 1 |
| Embase | ('pulmonary disease, chronic obstructive'/exp OR 'pulmonary disease, chronic obstructive' OR 'chronic obstructive pulmonary':ti,ab OR 'obstructive pulmonary disease*':ti,ab OR copd:ti,ab OR coad:ti,ab OR 'obstructive airway disease*':ti,ab OR 'chronic airflow obstruction*':ti,ab OR 'lung diseases, obstructive'/exp OR 'lung diseases, obstructive' OR 'obstructive lung disease*':ti,ab) AND ('periodontal diseases'/exp OR 'periodontal diseases' OR 'periodontal disease*':ti,ab OR parodontos*:ti,ab OR 'gingival diseases'/exp OR 'gingival diseases' OR 'gingival disease*':ti,ab OR gingivos*:ti,ab OR 'gingivitis'/exp OR gingivitis OR gingiviti*:ti,ab OR 'periodontitis'/exp OR periodontitis OR periodontiti*:ti,ab OR 'aggressive periodontitis'/exp OR 'aggressive periodontitis' OR 'chronic periodontitis'/exp OR 'chronic periodontitis' OR 'chronic periodontiti*':ti,ab OR 'periapical periodontitis'/exp OR 'periapical periodontitis' OR 'periodontal abscess'/exp OR 'periodontal abscess' OR 'periodontal abscess*':ti,ab OR 'periodontal pocket'/exp OR 'periodontal pocket' OR 'periodontal pocket*':ti,ab) AND ('systematic reviews as topic'/exp OR 'systematic reviews as topic' OR 'systematic review'/exp OR 'systematic review' OR term:it OR 'meta analysis as topic'/exp OR 'meta analysis as topic' OR 'meta analysis'/exp OR 'meta analysis') | 94 |
| Web of Science | (ALL="pulmonary disease, chronic obstructive" OR (TI="chronic obstructive pulmonary" OR AB="chronic obstructive pulmonary") OR (TI="obstructive pulmonary disease*" OR AB="obstructive pulmonary disease*") OR (TI=COPD OR AB=COPD) OR (TI=COAD OR AB=COAD) OR (TI="obstructive airway disease*" OR AB="obstructive airway disease*") OR (TI="chronic airflow obstruction*" OR AB="chronic airflow obstruction*") OR ALL="lung diseases, obstructive" OR (TI="obstructive lung disease*" OR AB="obstructive lung disease*")) AND (ALL="periodontal diseases" OR (TI="periodontal disease*" OR AB="periodontal disease*") OR (TI=parodontos* OR AB=parodontos*) OR ALL="gingival diseases" OR (TI="gingival disease*" OR AB="gingival disease*") OR (TI=gingivos* OR AB=gingivos*) OR ALL=gingivitis OR (TI=gingiviti* OR AB=gingiviti*) OR ALL=periodontitis OR (TI=periodontiti* OR AB=periodontiti*) OR ALL="aggressive periodontitis" OR ALL="chronic periodontitis" OR (TI="chronic periodontiti*" OR AB="chronic periodontiti*") OR ALL="periapical periodontitis" OR ALL="periodontal abscess" OR (TI="periodontal abscess*" OR AB="periodontal abscess*") OR ALL="periodontal pocket" OR (TI="periodontal pocket*" OR AB="periodontal pocket*")) AND (ALL="systematic review" OR ALL="systematic reviews as topic" OR ALL="systematic review" OR ALL="meta analysis" OR ALL="meta analysis as topic" OR ALL="meta analysis") | 16 |
| Scopus | (INDEXTERMS("pulmonary disease, chronic obstructive") OR TITLE-ABS("chronic obstructive pulmonary") OR TITLE-ABS("obstructive pulmonary disease*") OR TITLE-ABS(COPD) OR TITLE-ABS(COAD) OR TITLE-ABS("obstructive airway disease*") OR TITLE-ABS("chronic airflow obstruction*") OR INDEXTERMS("lung diseases, obstructive") OR TITLE-ABS("obstructive lung disease*")) AND (INDEXTERMS("periodontal diseases") OR TITLE-ABS("periodontal disease*") OR TITLE-ABS(parodontos*) OR INDEXTERMS("gingival diseases") OR TITLE-ABS("gingival disease*") OR TITLE-ABS(gingivos*) OR INDEXTERMS(gingivitis) OR TITLE-ABS(gingiviti*) OR INDEXTERMS(periodontitis) OR TITLE-ABS(periodontiti*) OR INDEXTERMS("aggressive periodontitis") OR INDEXTERMS("chronic periodontitis") OR TITLE-ABS("chronic periodontiti*") OR INDEXTERMS("periapical periodontitis") OR INDEXTERMS("periodontal abscess") OR TITLE-ABS("periodontal abscess*") OR INDEXTERMS("periodontal pocket") OR TITLE-ABS("periodontal pocket*")) AND (DOCTYPE("systematic review") OR INDEXTERMS("systematic reviews as topic") OR ALL("systematic review") OR DOCTYPE("meta analysis") OR INDEXTERMS("meta analysis as topic") OR ALL("meta analysis")) | 176 |
| Google Scholar | (("periodontal disease") OR ("periodontitis")) + ("chronic obstructive pulmonary diseases") OR (“COPD”) + (("systematic review") OR ("meta-analysis")) | 0 |
| Proquest | ("Periodontal Disease" OR “gingival disease” OR “periodontitis”) AND ("Chronic Obstructive Pulmonary Diseases ") AND (“systematic review” OR "meta-analysis") NOT ("animal" OR "in vitro" OR "tuberculosis" OR "neumonia" OR "knowledge") | 0 |
| OpenGrey | (("Periodontal Disease") OR ("furcation defect") OR ("gingival disease") OR ("periodontitis") OR (“periodontal therapy”) OR (“periodontal treatment”) OR (“root scaling”) OR (“periodontal debridement”) OR ("tooth migration") OR ("tooth mobility") OR ("tooth loss")) AND (("Respiratory tract diseases ") OR ("respiratory diseases") OR ("pulmonary disease") OR ("chronic obstructive pulmonary diseases") OR (“chronic bronchitis“) OR (“emphysema”) OR (“COPD”)) | 0 |
