# Supplementary material for: Association between periodontal disease and chronic obstructive pulmonary disease: an umbrella review
Source: Front Oral Health. 2026 Mar 27;7:1728405. doi: 10.3389/froh.2026.1728405 (PMC13066220; doi:10.3389/froh.2026.1728405)
Supplement: Supplementary file 2 [file Table2.docx]

Supplementary Material 2. Reason for exclusion of studies

| **Author(s)** | **Year** | **Reason for exclusion** |
| --- | --- | --- |
| Lin et al. (1) | 2023 | Literature review |
| Xiong et al. (2) | 2023 |  |

**References**

# Lin P, Liu A, Tsuchiya Y et al. Association between periodontal disease and chronic obstructive pulmonary disease. *Jpn Dent Sci Rev* (2023) 59: 389-402. doi:10.1016/j.jdsr.2023.10.004

# Xiong K, Yang P, Cui Y et al. Research on the Association Between Periodontitis and COPD. *Int J Chron Obstruct Pulmon Dis* (2023) 18: 1937-1948. doi:10.2147/COPD.S425172
