# Supplementary material for: Association between periodontal disease and chronic obstructive pulmonary disease: an umbrella review
Source: Front Oral Health. 2026 Mar 27;7:1728405. doi: 10.3389/froh.2026.1728405 (PMC13066220; doi:10.3389/froh.2026.1728405)
Supplement: Supplementary file 3 [file Table3.docx]

Supplementary Material 3. Characteristics of the included studies

| **Author(s)** | **Year** | **Study design** | **Country** | **Design of included studies** | **Number of studies in qualitative analysis** | **Number of studies in quantitative analysis** | **Results** | | **Conclusions** |
| --- | --- | --- | --- | --- | --- | --- | --- | --- | --- |
| Molina et al. (1) | 2023 | SR and MA | Spain | CC, C and CS | 34 | 12 | COPD | OR = 1.28 (1.16 – 1.42) | There is an association between PE and COPD. |
| Yang et al. (2) | 2023 | SR and MA | China | CC, C and CS | 22 | 18 | COPD | OR = 1.2 (1.09 – 1.32) | PD was weakly associated with COPD risk. Furthermore, it did not increase the risk of COPD exacerbations or mortality, regardless of smoking status. |
|  |  |  |  |  |  |  | ABL | OR = 1.98 (1.32 – 2.97) |  |
|  |  |  |  |  |  |  | COPD intensity | OR = 1.14 (0.86 – 1.51) |  |
|  |  |  |  |  |  |  | Smokers | OR = 1.46 (0.92 – 2.31) |  |
|  |  |  |  |  |  |  | Non-Smokers | OR = 0.93 (0.72 – 1.21) |  |
|  |  |  |  |  |  |  | COPD exacerbations | OR = 1.18 (0.71 – 1.21) |  |
| Wu et al. (3) | 2022 | SR and MA | China | CC, C and CS | 22 | 20 | COPD | OR = 1.64 (1.19 – 2.27) | There is an association between PD and COPD. |
|  |  |  |  |  |  |  | OHI | WMD = 0.81 (0.48 – 1.14) |  |
|  |  |  |  |  |  |  | RT | WMD = -3.51 (-4.66 – -2.35) |  |
|  |  |  |  |  |  |  | ABL | WMD = 0.63 (0.26 – 0.99) |  |
|  |  |  |  |  |  |  | PD | WMD = 0.33 (0.11 – 0.55) |  |
|  |  |  |  |  |  |  | CLA | WMD = 0.69 (0.45 – 0.93) |  |
|  |  |  |  |  |  |  | PI | WMD = 0.29 (0.11 – 0.47) |  |
|  |  |  |  |  |  |  | IG | WMD = 0.41 (0.12 – 0.7) |  |
| Kelly et al. (4) | 2021 | SR | United Kingdom | CC, C, CS and CT | 8 | 0 | COPD exacerbations | There is a possible positive correlation between improved periodontal health, reduced hospitalizations, and improved quality of life in patients with COPD. | Poor periodontal health and poor oral hygiene are associated with COPD exacerbations. |
| Gomes-Filho et al. (5) | 2020 | SR and MA | Brazil | CC, C and CS | 7 | 4 | COPD | OR = 1.78 (1.04 – 3.05) | There is a moderate association between PD and COPD. |
| Mushtaq et al. (6) | 2019 | SR | Pakistan | CC and CS | 4 | 0 | COPD | A weak association was identified in four studies between PD and COPD. | There is a weak association between COPD and PD. |
| Shi et al. (7) | 2018 | SR and MA | China | CC and CS | 14 | 12 | OHI | MD = 0.8 (0.33 – 1.28) | Patients with COPD suffer from poorer periodontal health, indicated by deeper periodontal pockets, high level of clinical attachment loss, poorer oral hygiene, more inflammation and bleeding in the gingival tissue, and fewer remaining teeth. |
|  |  |  |  |  |  |  | RT | MD = -3.73 (-5.12 – -2.33) |  |
|  |  |  |  |  |  |  | ABL | MD = 0.13 (0.00 – 0.25) |  |
|  |  |  |  |  |  |  | PD | MD = 0.26 (0.02 – 0.5) |  |
|  |  |  |  |  |  |  | CLA | MD = 0.48 (0.28 – 0.68) |  |
|  |  |  |  |  |  |  | PI | MD = 0.23 (0.04 – 0.41) |  |
|  |  |  |  |  |  |  | GI | MD = 0.36 (0.04 – 0.69) |  |
|  |  |  |  |  |  |  | BI | MD = 0.24 (-0.11 – 0.59) |  |
|  |  |  |  |  |  |  | BP | MD = 6.88 (5.49 – 8.27) |  |
| Tan et al. (8) | 2016 | SR and MA | China | CC and CS | 5 | 5 | Non-smoking CLA | OR = 1.0 (0.99 – 1.01) | CLA, BI, or PD did not increase the risk of COPD in nonsmokers, current smokers, or ex-smokers. There may be an interaction between PI and smoking in the development of COPD. |
|  |  |  |  |  |  |  | Ex-smokers CLA | OR = 1.39 (0.77 – 2.5) |  |
|  |  |  |  |  |  |  | Smokers CLA | OR = 0.99 (0.98 – 1.0) |  |
|  |  |  |  |  |  |  | Non-smoking PI | OR = 1.52 (0.76 – 3.05) |  |
|  |  |  |  |  |  |  | Ex-smokers PI | OR = 2.18 (0.89 – 5.33) |  |
|  |  |  |  |  |  |  | Smokers PI | OR = 3.99 (2.58 – 6.16) |  |
|  |  |  |  |  |  |  | Non-smoking BI | OR = 1.19 (0.58 – 2.46) |  |
|  |  |  |  |  |  |  | Ex-smokers BI | OR = 0.37 (0.13 – 1.01) |  |
|  |  |  |  |  |  |  | Smokers BI | OR = 1.35 (0.57 – 3.24) |  |
|  |  |  |  |  |  |  | Non-smoking PD | OR = 0.3 (0.15 – 0.62) |  |
|  |  |  |  |  |  |  | Smokers PD | OR = 0.43 (0.14 – 1.31) |  |
| Zeng et al. (9) | 2012 | SR and MA | China | CC and CS | 14 | 14 | COPD | OR = 2.08 (1.48 – 2.91) | PD is a significant and independent risk factor for COPD. |
| Azarpazhooh et al. (10) | 2006 | SR | Canada | CC and CS | 4 | 0 | COPD | A weak association was identified between PD and COPD. | There is a weak association between PD and COPD. |
| Scannapieco et al. (11) | 2003 | SR | USA | CC and CS | 4 | 0 | COPD | Poor oral hygiene and PD are associated with respiratory diseases such as COPD. | There is a possible association between PD and COPD. |
| Garcia et al. (12) | 2001 | SR | USA | CC, CS and CT | 6 | 0 | COPD | Poorer periodontal health increases the risk of developing COPD. | There is a causal association between periodontal health status and the risk of COPD. |

PD = Periodontal disease; COPD = Chronic obstructive pulmonary disease; SR = Systematic review; MA = Meta-analysis; CC = Case-control; C = Cohorts; CS = Cross-sectional; CT = Clinical trial; ABL = Alveolar bone loss; OHI = Oral hygiene index; PD = Probing depth; PI = Plaque index; GI = Gingival index; RT = Remaining teeth; CLA = Clinical level and attachment; BI = Bleeding index; BP = Bleeding on probing

**References**

# Molina A, Huck O, Herrera D et al. The association between respiratory diseases and periodontitis: A systematic review and meta-analysis. *J Clin Periodontol* (2023) 50(6): 842-887. doi:10.1111/jcpe.13767.

# Yang M, Peng R, Li X et al. Association between chronic obstructive pulmonary disease and periodontal disease: a systematic review and meta-analysis. *BMJ Open* (2023) 13(6): e067432. doi:10.1136/bmjopen-2022-067432

# Wu Z, Xiao C, Chen F et al. Pulmonary disease and periodontal health: a meta-analysis. *Sleep Breath Schlaf Atm* (2022) 26(4): 1857-1868. doi:10.1007/s11325-022-02577-3.

# Kelly N, Winning L, Irwin C et al. Periodontal status and chronic obstructive pulmonary disease (COPD) exacerbations: a systematic review. *BMC Oral Health* (2021) 21(1): 425. doi:10.1186/s12903-021-01757-z

# Gomes-Filho IS, Cruz SS da, Trindade SC et al. Periodontitis and respiratory diseases: A systematic review with meta-analysis. *Oral Dis* (2020) 26(2): 439-446. doi:10.1111/odi.13228

# Mushtaq S, Ammaar M, Sajjad E. Association between respiratory diseases and oral health: A systematic review study. *Indo Am J Pharm Sci* (2019) 6(5): 10800-10807.

# Shi Q, Zhang B, Xing H et al. Patients with Chronic Obstructive Pulmonary Disease Suffer from Worse Periodontal Health-Evidence from a Meta-Analysis. *Front Physiol* (2018) 9:33. doi: 10.3389/fphys.2018.00033

# Tan L, Wang H, Pan C et al. Periodontal health and chronic obstructive pulmonary disease stratified by smoking: a meta-analysis. *Int J Clin Exp Med* (2016) 9(12): 23190-23197.

# Zeng XT, Tu ML, Liu DY et al. Periodontal disease and risk of chronic obstructive pulmonary disease: a meta-analysis of observational studies. *PloS One* (2012) 7(10): e46508. doi:10.1371/journal.pone.0046508

# Azarpazhooh A, Leake JL. Systematic review of the association between respiratory diseases and oral health. *J Periodontol* (2006) 77(9): 1465-1482. doi:10.1902/jop.2006.060010.

# Scannapieco FA, Bush RB, Paju S. Associations between periodontal disease and risk for nosocomial bacterial pneumonia and chronic obstructive pulmonary disease. A systematic review. *Ann Periodontol* (2003) 8(1): 54-69. doi:10.1902/anales.2003.8.1.54.

# Garcia RI, Nunn ME, Vokonas PS. Epidemiologic associations between periodontal disease and chronic obstructive pulmonary disease. *Ann Periodontol* (2001) 6(1): 71-77. doi:10.1902/anales.2001.6.1.71.
