# Supplementary material for: Association between periodontal disease and chronic obstructive pulmonary disease: an umbrella review
Source: Front Oral Health. 2026 Mar 27;7:1728405. doi: 10.3389/froh.2026.1728405 (PMC13066220; doi:10.3389/froh.2026.1728405)
Supplement: Supplementary file 4 [file Table4.docx]

Supplementary Material 4. Assessment of methodological and evidence quality of the included studies

| **Authors** | **Year** | **AMSTAR – 2** | | | | | | | | | | | | | | | | **General confidence** |
| --- | --- | --- | --- | --- | --- | --- | --- | --- | --- | --- | --- | --- | --- | --- | --- | --- | --- | --- |
|  |  | 1 | 2* | 3 | 4* | 5 | 6 | 7* | 8 | 9* | 10 | 11* | 12 | 13* | 14 | 15* | 16 |  |
| Molina et al. (1) | 2023 | Y | Y | Y | Y | Y | Y | Y | Y | Y | Y | Y | Y | Y | Y | Y | Y | High |
| Yang et al. (2) | 2023 | Y | Y | Y | Y | Y | Y | Y | Y | Y | Y | Y | Y | Y | Y | Y | Y | High |
| Wu et al. (3) | 2022 | Y | Y | Y | Y | Y | Y | Y | Y | Y | Y | Y | Y | Y | Y | Y | Y | High |
| Kelly et al. (4) | 2021 | Y | Y | Y | Y | Y | Y | Y | Y | Y | Y | NMA | | Y | Y | NMA | Y | High |
| Gomes-Filho et al. (5) | 2020 | Y | Y | Y | Y | Y | Y | Y | Y | Y | Y | S |  | Y | Y | Y | Y | High |
| Mushtaq et al. (6) | 2019 | Y | N | Y | N | N | N | N | Y | N | Y | NMA | | N | N | NMA | N | Critically low |
| Shi et al. (7) | 2018 | Y | Y | Y | Y | Y | Y | Y | Y | Y | Y | Y | Y | Y | Y | Y | Y | High |
| Tan et al. (8) | 2016 | Y | N | Y | Y | Y | N | N | Y | N | Y | Y | Y | N | Y | Y | Y | Critically low |
| Zeng et al. (9) | 2012 | Y | Y | Y | Y | Y | Y | Y | Y | N | Y | Y | Y | N | Y | Y | Y | Critically low |
| Azarpazhooh et al. (10) | 2006 | Y | N | Y | Y | N | N | Y | Y | SP | Y | NMA | | Y | Y | NMA | N | Low |
| Scannapieco et al. (11) | 2003 | Y | N | Y | Y | Y | N | N | Y | N | Y | NMA | | N | Y | NMA | N | Critically low |
| Garcia et al. (12) | 2001 | Y | N | Y | N | N | N | N | Y | SP | Y | NMA | | Y | Y | NMA | N | Critically low |

AMSTAR = A Measurement Tool for Assessing Systematic Reviews; 1 = Did the research questions and inclusion criteria for the review include the components of PICO?; 2 = Did the review report contain an explicit statement that the review methods were established prior to the conduct of the review, and did the report justify any significant departures from the protocol?; 3 = Did the review authors explain their selection of study designs for inclusion in the review?; 4 = Did the review authors use a comprehensive literature search strategy?; 5 = Did the review authors perform study selection in duplicate?; 6 = Did the review authors perform data extraction in duplicate?; 7 = Did the review authors provide a list of excluded studies and justify the exclusions?; 8 = Did the review authors describe the included studies in adequate detail?; 9 = Did the review authors use a satisfactory technique to assess the risk of bias (RoB) in the individual studies included in the review? 10 = Did the review authors report the funding sources of the studies included in the review?; 11 = If a meta-analysis was performed, did the review authors use appropriate methods for statistical pooling of results?; 12 = If a meta-analysis was performed, did the review authors assess the potential impact of publication bias (ROP) in individual studies on the results of the meta-analysis or other evidence synthesis?; 13 = Did the review authors consider ROP in the primary studies when interpreting or analyzing the review results?; 14 = Did the review authors provide a satisfactory explanation for and discuss any observed heterogeneity in the review results?; 15 = If a quantitative synthesis was performed, did the review authors adequately investigate publication bias (small study bias) and discuss its likely impact on the review results? 16 = Did the review authors disclose potential sources of conflict of interest, including funding received for the review?; Y = Yes; N = No; SP = Partial Yes; NMA = No meta-analysis; * = Critical domain

**References**

# Molina A, Huck O, Herrera D et al. The association between respiratory diseases and periodontitis: A systematic review and meta-analysis. *J Clin Periodontol* (2023) 50(6): 842-887. doi:10.1111/jcpe.13767.

# Yang M, Peng R, Li X et al. Association between chronic obstructive pulmonary disease and periodontal disease: a systematic review and meta-analysis. *BMJ Open* (2023) 13(6): e067432. doi:10.1136/bmjopen-2022-067432

# Wu Z, Xiao C, Chen F et al. Pulmonary disease and periodontal health: a meta-analysis. *Sleep Breath Schlaf Atm* (2022) 26(4): 1857-1868. doi:10.1007/s11325-022-02577-3.

# Kelly N, Winning L, Irwin C et al. Periodontal status and chronic obstructive pulmonary disease (COPD) exacerbations: a systematic review. *BMC Oral Health* (2021) 21(1): 425. doi:10.1186/s12903-021-01757-z

# Gomes-Filho IS, Cruz SS da, Trindade SC et al. Periodontitis and respiratory diseases: A systematic review with meta-analysis. *Oral Dis* (2020) 26(2): 439-446. doi:10.1111/odi.13228

# Mushtaq S, Ammaar M, Sajjad E. Association between respiratory diseases and oral health: A systematic review study. *Indo Am J Pharm Sci* (2019) 6(5): 10800-10807.

# Shi Q, Zhang B, Xing H et al. Patients with Chronic Obstructive Pulmonary Disease Suffer from Worse Periodontal Health-Evidence from a Meta-Analysis. *Front Physiol* (2018) 9:33. doi: 10.3389/fphys.2018.00033

# Tan L, Wang H, Pan C et al. Periodontal health and chronic obstructive pulmonary disease stratified by smoking: a meta-analysis. *Int J Clin Exp Med* (2016) 9(12): 23190-23197.

# Zeng XT, Tu ML, Liu DY et al. Periodontal disease and risk of chronic obstructive pulmonary disease: a meta-analysis of observational studies. *PloS One* (2012) 7(10): e46508. doi:10.1371/journal.pone.0046508

# Azarpazhooh A, Leake JL. Systematic review of the association between respiratory diseases and oral health. *J Periodontol* (2006) 77(9): 1465-1482. doi:10.1902/jop.2006.060010.

# Scannapieco FA, Bush RB, Paju S. Associations between periodontal disease and risk for nosocomial bacterial pneumonia and chronic obstructive pulmonary disease. A systematic review. *Ann Periodontol* (2003) 8(1): 54-69. doi:10.1902/anales.2003.8.1.54.

# Garcia RI, Nunn ME, Vokonas PS. Epidemiologic associations between periodontal disease and chronic obstructive pulmonary disease. *Ann Periodontol* (2001) 6(1): 71-77. doi:10.1902/anales.2001.6.1.71.
