# Supplementary material for: Association between periodontal disease and chronic obstructive pulmonary disease: an umbrella review
Source: Front Oral Health. 2026 Mar 27;7:1728405. doi: 10.3389/froh.2026.1728405 (PMC13066220; doi:10.3389/froh.2026.1728405)
Supplement: Supplementary file 5 [file Table5.docx]

Supplementary Material 5. GRADE assessment of the included studies

| **Autor(s)** | **GRADE** |
| --- | --- |
| Molina, A. et al. (1) | Yes, moderate |
| Yang, M. et al. (2) | None |
| Wu, Z. et al. (3) | None |
| Kelly N. et al. (4) | None |
| Gomes-Filho, I. et al. (5) | None |
| Mushtaq, S. et al. (6) | None |
| Shi, Q. et al. (7) | None |
| Tan, L. et al. (8) | None |
| Zeng, X. et al. (9) | None |
| Azarpazhooh, A. et al. (10) | None |
| Scannapieco, F. et al. (11) | None |
| Garcia, R. et al. (12) | None |

**References**

# Molina A, Huck O, Herrera D et al. The association between respiratory diseases and periodontitis: A systematic review and meta-analysis. *J Clin Periodontol* (2023) 50(6): 842-887. doi:10.1111/jcpe.13767.

# Yang M, Peng R, Li X et al. Association between chronic obstructive pulmonary disease and periodontal disease: a systematic review and meta-analysis. *BMJ Open* (2023) 13(6): e067432. doi:10.1136/bmjopen-2022-067432

# Wu Z, Xiao C, Chen F et al. Pulmonary disease and periodontal health: a meta-analysis. *Sleep Breath Schlaf Atm* (2022) 26(4): 1857-1868. doi:10.1007/s11325-022-02577-3.

# Kelly N, Winning L, Irwin C et al. Periodontal status and chronic obstructive pulmonary disease (COPD) exacerbations: a systematic review. *BMC Oral Health* (2021) 21(1): 425. doi:10.1186/s12903-021-01757-z

# Gomes-Filho IS, Cruz SS da, Trindade SC et al. Periodontitis and respiratory diseases: A systematic review with meta-analysis. *Oral Dis* (2020) 26(2): 439-446. doi:10.1111/odi.13228

# Mushtaq S, Ammaar M, Sajjad E. Association between respiratory diseases and oral health: A systematic review study. *Indo Am J Pharm Sci* (2019) 6(5): 10800-10807.

# Shi Q, Zhang B, Xing H et al. Patients with Chronic Obstructive Pulmonary Disease Suffer from Worse Periodontal Health-Evidence from a Meta-Analysis. *Front Physiol* (2018) 9:33. doi: 10.3389/fphys.2018.00033

# Tan L, Wang H, Pan C et al. Periodontal health and chronic obstructive pulmonary disease stratified by smoking: a meta-analysis. *Int J Clin Exp Med* (2016) 9(12): 23190-23197.

# Zeng XT, Tu ML, Liu DY et al. Periodontal disease and risk of chronic obstructive pulmonary disease: a meta-analysis of observational studies. *PloS One* (2012) 7(10): e46508. doi:10.1371/journal.pone.0046508

# Azarpazhooh A, Leake JL. Systematic review of the association between respiratory diseases and oral health. *J Periodontol* (2006) 77(9): 1465-1482. doi:10.1902/jop.2006.060010.

# Scannapieco FA, Bush RB, Paju S. Associations between periodontal disease and risk for nosocomial bacterial pneumonia and chronic obstructive pulmonary disease. A systematic review. *Ann Periodontol* (2003) 8(1): 54-69. doi:10.1902/anales.2003.8.1.54.

# Garcia RI, Nunn ME, Vokonas PS. Epidemiologic associations between periodontal disease and chronic obstructive pulmonary disease. *Ann Periodontol* (2001) 6(1): 71-77. doi:10.1902/anales.2001.6.1.71.
