# Supplementary material for: Association between periodontal disease and chronic obstructive pulmonary disease: an umbrella review
Source: Front Oral Health. 2026 Mar 27;7:1728405. doi: 10.3389/froh.2026.1728405 (PMC13066220; doi:10.3389/froh.2026.1728405)
Supplement: Supplementary file 6 [file Table6.docx]

Supplementary Material 6. Overlap of primary studies included in systematic reviews

| **Authors** | **Systematic review** | **Times repeated** |
| --- | --- | --- |
| Scannapieco et al. (1) | Molina et al. (2), Yang et al. (3), Wu et al. (4), Mushtaq et al. (5), Shi et al. (6), Zeng et al. (7), Azarpazhooh et al. (8), Scannapieco et al. (9), Garcia et al. (10) | 9 |
| Scannapieco et al. (11) | Molina et al. (2), Yang et al. (3), Wu et al. (4), Mushtaq et al. (5), Zeng et al. (7), Azarpazhooh et al. (8), Scannapieco et al. (9), Garcia et al. (10) | 8 |
| Hayes et al. (12) | Yang et al. (3), Wu et al. (4), Mushtaq et al. (5), Shi et al. (6), Zeng et al. (7), Azarpazhooh et al. (8), Scannapieco et al. (9), Garcia et al. (10) | 8 |
| Si et al. (13) | Molina et al. (2), Yang et al. (3), Wu et al. (4), Gomes-Filho et al. (14), Shi et al. (6), Tan et al. (15), Zeng et al. (7) | 7 |
| Wang et al. (16) | Molina et al. (2), Yang et al. (3), Wu et al. (4), Shi et al. (6), Tan et al. (15), Zeng et al. (7) | 6 |
| Hyman et al. (17) | Molina et al. (2), Yang et al. (3), Wu et al. (4), Tan et al. (15), Zeng et al. (7) | 5 |
| Deo et al. (18) | Molina et al. (2), Wu et al. (4), Shi et al. (6), Tan et al. (15), Zeng et al. (7) | 5 |
| Ledić et al. (19) | Molina et al. (2), Yang et al. (3), Wu et al. (4), Gomes-Filho et al. (14), Shi et al. (6) | 5 |
| Barros et al. (20) | Molina et al. (2), Yang et al. (3), Wu et al. (4), Kelly et al. (21), Gomes-Filho et al. (14) | 5 |
| Chung et al. (22) | Molina et al. (2), Yang et al. (3), Wu et al. (4), Gomes-Filho et al. (14), Shi et al. (6) | 5 |
| Prasanna (23) | Molina et al. (2), Wu et al. (4), Shi et al. (6), Zeng et al. (7) | 4 |
| Terashima et al. (24) | Molina et al. (2), Wu et al. (4), Gomes-Filho et al. (14), Shi et al. (6) | 4 |
| Harland et al. (25) | Molina et al. (2), Yang et al. (3), Wu et al. (4), Gomes-Filho et al. (14) | 4 |
| Takeuchi et al. (26) | Molina et al. (2), Yang et al. (3), Wu et al. (4), Gomes-Filho et al. (14) | 4 |
| Russell et al. (27) | Mushtaq et al. (5), Zeng et al. (7), Azarpazhooh et al. (8) | 3 |
| Garcia et al. (10) | Yang et al. (3), Zeng et al. (7), Scannapieco et al. (9) | 3 |
| Leuckfeld et al. (28) | Yang et al. (3), Wu et al. (4), Zeng et al. (7) | 3 |
| Zhou et al. (29) | Yang et al. (3), Tan et al. (15), Zeng et al. (7) | 3 |
| Liu et al. (30) | Molina et al. (2), Yang et al. (3), Kelly et al. (21) | 3 |
| Peter et al. (31) | Molina et al. (2), Wu et al. (4), Shi et al. (6) | 3 |
| Yildirim et al. (32) | Molina et al. (2), Wu et al. (4), Shi et al. (6) | 3 |
| Öztekin et al. (33) | Molina et al. (2), Wu et al. (4), Shi et al. (6) | 3 |
| Vadiraj et al. (34) | Wu et al. (4), Shi et al. (6) | 2 |
| Bhavsar et al. (35) | Wu et al. (4), Shi et al. (6) | 2 |
| AbdelHalim et al. (36) | Yang et al. (3), Kelly et al. (21) | 2 |
| Tan et al. (37) | Molina et al. (2), Wu et al. (4) | 2 |
| Baldomero et al. (38) | Molina et al. (2), Kelly et al. (21) | 2 |
| Winning et al. (39) | Molina et al. (2), Yang et al. (3) | 2 |
| Jung et al. (40) | Molina et al. (2), Yang et al. (3) | 2 |

**References**

1 Scannapieco FA, Ho AW. Potential associations between chronic respiratory disease and periodontal disease: analysis of National Health and Nutrition Examination Survey III. *J Periodontol* (2001) 72(1): 50-56. doi:10.1902/jop.2001.72.1.50

2 Molina A, Huck O, Herrera D et al. The association between respiratory diseases and periodontitis: A systematic review and meta-analysis. *J Clin Periodontol* (2023) 50(6): 842-887. doi:10.1111/jcpe.13767.

3 Yang M, Peng R, Li X et al. Association between chronic obstructive pulmonary disease and periodontal disease: a systematic review and meta-analysis. *BMJ Open* (2023) 13(6): e067432. doi:10.1136/bmjopen-2022-067432

4 Wu Z, Xiao C, Chen F et al. Pulmonary disease and periodontal health: a meta-analysis. *Sleep Breath Schlaf Atm* (2022) 26(4): 1857-1868. doi:10.1007/s11325-022-02577-3.

5 Mushtaq S, Ammaar M, Sajjad E. Association between respiratory diseases and oral health: A systematic review study. *Indo Am J Pharm Sci* (2019) 6(5): 10800-10807. doi:10.5281/zenodo.3229308

6 Shi Q, Zhang B, Xing H et al. Patients with Chronic Obstructive Pulmonary Disease Suffer from Worse Periodontal Health-Evidence from a Meta-Analysis. *Front Physiol* (2018) 9:33. doi: 10.3389/fphys.2018.00033

7 Zeng XT, Tu ML, Liu DY et al. Periodontal disease and risk of chronic obstructive pulmonary disease: a meta-analysis of observational studies. *PloS One* (2012) 7(10): e46508. doi:10.1371/journal.pone.0046508

8 Azarpazhooh A, Leake JL. Systematic review of the association between respiratory diseases and oral health. *J Periodontol* (2006) 77(9): 1465-1482. doi:10.1902/jop.2006.060010

9 Scannapieco FA, Bush RB, Paju S. Associations between periodontal disease and risk for nosocomial bacterial pneumonia and chronic obstructive pulmonary disease. A systematic review. *Ann Periodontol* (2003) 8(1): 54-69. doi:10.1902/anales.2003.8.1.54.

10 Garcia RI, Nunn ME, Vokonas PS. Epidemiologic associations between periodontal disease and chronic obstructive pulmonary disease. *Ann Periodontol* (2001) 6(1): 71-77. doi:10.1902/anales.2001.6.1.71

11 Scannapieco FA, Papandonatos GD, Dunford RG. Associations between oral conditions and respiratory disease in a national sample survey population. *Ann Periodontol* (1998) 3(1): 251-256. doi: 10.1902/anales.1998.3.1.251

12 Hayes C, Sparrow D, Cohen M et al. The association between alveolar bone loss and pulmonary function: the VA Dental Longitudinal Study. *Ann Periodontol* (1998) 3(1): 257-261. doi: 10.1902/anales.1998.3.1.257

13 Si Y, Fan H, Song Y et al. Association between periodontitis and chronic obstructive pulmonary disease in a Chinese population. *J Periodontol* (2012) 83(10): 1288-1296. doi:

14 Gomes-Filho IS, Cruz SS da, Trindade SC et al. Periodontitis and respiratory diseases: A systematic review with meta-analysis. *Oral Dis* (2020) 26(2): 439-446. doi:10.1111/odi.13228

15 Tan L, Wang H, Pan C et al. Periodontal health and chronic obstructive pulmonary disease stratified by smoking: a meta-analysis. *Int J Clin Exp Med* (2016) 9(12): 23190-23197.

16 Wang Z, Zhou X, Zhang J et al. Periodontal health, oral health behaviours, and chronic obstructive pulmonary disease. *J Clin Periodontol* (2009) 36(9): 750-755. doi:10.1111/j.1600-051X.2009.01448.x

17 Hyman JJ, Reid BC. Cigarette smoking, periodontal disease: and chronic obstructive pulmonary disease. *J Periodontol* (2004) 75(1): 9-15. doi:10.1902/jop.2004.75.1.9

18 Deo V, Bhongade ML, Ansari S et al. Periodontitis as a potential risk factor for chronic obstructive pulmonary disease: a retrospective study. *Indian J Dent Res Off Publ Indian Soc Dent* *Res* (2009) 20(4): 466-470. doi: 10.4103/0970-9290.59456

19 Ledić K, Marinković S, Puhar I et al. Periodontal disease increases risk for chronic obstructive pulmonary disease. *Coll Antropol* (2013) 37(3): 937-942.

20 Barros SP, Suruki R, Loewy ZG et al. A cohort study of the impact of tooth loss and periodontal disease on respiratory events among COPD subjects: modulatory role of systemic biomarkers of inflammation. *PloS One* (2013) 8(8): e68592. doi: 10.1371/journal.pone.0068592.

21 Kelly N, Winning L, Irwin C et al. Periodontal status and chronic obstructive pulmonary disease (COPD) exacerbations: a systematic review. *BMC Oral Health* (2021) 21(1): 425. doi:10.1186/s12903-021-01757-z

22 Chung JH, Hwang HJ, Kim SH et al. Associations Between Periodontitis and Chronic Obstructive Pulmonary Disease: The 2010 to 2012 Korean National Health and Nutrition Examination Survey. *J Periodontol* (2016) 87(8): 864-871. doi:10.1902/jop.2016.150682

23 Prasanna SJ. Causal relationship between periodontitis and chronic obstructive pulmonary disease. *J Indian Soc Periodontol* (2011) 15(4): 359-365. doi:10.4103/0972-124X.92570

24 Terashima T, Chubachi S, Matsuzaki T et al. The association between dental health and nutritional status in chronic obstructive pulmonary disease. *Chron Respir Dis* (2017) 14(4): 334-341. doi:10.1177/1479972316643076

25 Harland J, Furuta M, Takeuchi K et al. Periodontitis modifies the association between smoking and chronic obstructive pulmonary disease in Japanese men. *J Oral Sci* (2018) 60(2): 226-231. doi:10.2334/josnusd.17-0225

26 Takeuchi K, Matsumoto K, Furuta M et al. Periodontitis Is Associated with Chronic Obstructive Pulmonary Disease. *J Dent Res* (2019) 98(5): 534-540. doi:10.1177/0022034519833630

27 Russell SL, Boylan RJ, Kaslick RS et al. Respiratory pathogen colonization of the dental plaque of institutionalized elders. *Spec Care Dent Off Publ Am Assoc Hosp Dent Acad Dent Handicap Am Soc Geriatr Dent* (1999) 19(3): 128-134. doi:10.1111/j.1754-4505.1999.tb01413.x

28 Leuckfeld I, Obregon-Whittle MV, Lund MB et al. Severe chronic obstructive pulmonary disease: association with marginal bone loss in periodontitis. *Respir Med* (2008) 102(4): 488-494. doi:10.1016/j.rmed.2007.12.001

29 Zhou X, Han J, Song Y et al. Serum levels of 25-hydroxyvitamin D, oral health and chronic obstructive pulmonary disease. *J Clin Periodontol* (2012) 39(4): 350-356. doi:10.1111/j.1600-051X.2012.01852.x

30 Liu Z, Zhang W, Zhang J et al. Oral hygiene, periodontal health and chronic obstructive pulmonary disease exacerbations. *J Clin Periodontol* (2012) 39(1): 45-52. doi:10.1111/j.1600-051X.2011.01808.x

31 Peter KP, Mute BR, Doiphode SS et al. Association between periodontal disease and chronic obstructive pulmonary disease: a reality or just a dogma? *J Periodontol* (2013) 84(12): 1717-1723. doi:10.1902/jop.2013.120347

32 Yıldırım E, Kormi I, Başoğlu ÖK et al. Periodontal health and serum, saliva matrix metalloproteinases in patients with mild chronic obstructive pulmonary disease. *J Periodontal Res* (2013) 48(3): 269-275. doi:10.1111/jre.12004

33 Öztekin G, Baser U, Kucukcoskun M et al. The association between periodontal disease and chronic obstructive pulmonary disease: a case control study. *COPD* (2014) 11(4): 424-430. doi:10.3109/15412555.2013.858316

34 Vadiraj S, Nayak R, Choudhary GK et al. Periodontal pathogens and respiratory diseases- evaluating their potential association: a clinical and microbiological study. *J Contemp Dent Pract* (2013) 14(4): 610-615. doi:10.5005/jp-journals-10024-1373

35 Bhavsar NV, Dave BD, Brahmbhatt NA et al. Periodontal status and oral health behavior in hospitalized patients with chronic obstructive pulmonary disease. *J Nat Sci Biol Med* (2015) 6(Suppl 1): S93-97. doi:10.4103/0976-9668.166097

36 AbdelHalim HA, AboElNaga HH, Aggour RL. Chronic obstructive pulmonary disease exacerbations and periodontitis: a possible association. *Egypt J Bronchol* (2018) 12(3): 303-309. doi:10.4103/ejb.ejb_12_18.

37 Tan L, Tang X, Pan C et al. Relationship among clinical periodontal, microbiologic parameters and lung function in participants with chronic obstructive pulmonary disease. *J Periodontol* (2019) 90(2): 134-140. doi:10.1002/JPER.17-0705.

38 Baldomero AK, Siddiqui M, Lo CY et al. The relationship between oral health and COPD exacerbations. *Int J Chron Obstruct Pulmon Dis* (2019) 14: 881-892. doi:10.2147/COPD.S194991

39 Winning L, Polyzois I, Sanmartin J et al. Periodontitis and airflow limitation in older Swedish individuals. *J Clin Periodontol* (2020) 47(6): 715-725. doi:10.1111/jcpe.13287.

40 Jung ES, Lee KH, Choi YY. Association between oral health status and chronic obstructive pulmonary disease in Korean adults. *Int Dent J* (2020) 70(3): 208-213. doi:10.1111/idj.12535.
