# Supplementary material for: Association between periodontal disease and chronic obstructive pulmonary disease: an umbrella review
Source: Front Oral Health. 2026 Mar 27;7:1728405. doi: 10.3389/froh.2026.1728405 (PMC13066220; doi:10.3389/froh.2026.1728405)
Supplement: Supplementary file 7 [file Table7.docx]

Supplementary Material 7. Synthesis of Results of Included Studies

| **Authors** | **Results** | | **Association** |
| --- | --- | --- | --- |
| Molina et al. (1) | COPD | OR = 1.28 (1.16 – 1.42) | Yes |
| Yang et al. (2) | COPD | OR = 1.2 (1.09 – 1.32) | Yes |
|  | ABL | OR = 1.98 (1.32 – 2.97) | Yes |
|  | Smoking intensity | OR = 1.14 (0.86 – 1.51) | No |
|  | Smokers | OR = 1.46 (0.92 – 2.31) | No |
|  | Non-smokers | OR = 0.93 (0.72 – 1.21) | No |
|  | COPD exacerbations | OR = 1.18 (0.71 – 1.21) | No |
| Wu et al. (3) | COPD | OR = 1.64 (1.19 – 2.27) | Yes |
|  | OHI | WMD = 0.81 (0.48 – 1.14) | Yes |
|  | RT | WMD = -3.51 (-4.66 – -2.35) | Yes |
|  | ABL | WMD = 0.63 (0.26 – 0.99) | Yes |
|  | PD | WMD = 0.33 (0.11 – 0.55) | Yes |
|  | CLA | WMD = 0.69 (0.45 – 0.93) | Yes |
|  | PI | WMD = 0.29 (0.11 – 0.47) | Yes |
|  | GI | WMD = 0.41 (0.12 – 0.7) | Yes |
| Kelly et al. (4) | COPD exacerbations | There is a possible positive correlation between improved periodontal health, reduced hospitalizations, and improved quality of life in patients with COPD. | Yes |
| Gomes-Filho et al. (5) | COPD | OR = 1.78 (1.04 – 3.05) | Yes |
| Mushtaq et al. (6) | COPD | A weak association was identified in four studies between PD and COPD. | Yes |
| Shi et al. (7) | OHI | MD = 0.8 (0.33 – 1.28) | Yes |
|  | RT | MD = -3.73 (-5.12 – -2.33) | Yes |
|  | ABL | MD = 0.13 (0.00 – 0.25) | No |
|  | PD | MD = 0.26 (0.02 – 0.5) | Yes |
|  | CLA | MD = 0.48 (0.28 – 0.68) | Yes |
|  | PI | MD = 0.23 (0.04 – 0.41) | Yes |
|  | GI | MD = 0.36 (0.04 – 0.69) | Yes |
|  | BI | MD = 0.24 (-0.11 – 0.59) | No |
|  | BP | MD = 6.88 (5.49 – 8.27) | Yes |
| Tan et al. (8) | Non-smokers CLA | OR = 1.0 (0.99 – 1.01) | No |
|  | Ex-smokers CLA | OR = 1.39 (0.77 – 2.5) | No |
|  | Smokers CLA | OR = 0.99 (0.98 – 1.0) | No |
|  | Non-smokers PI | OR = 1.52 (0.76 – 3.05) | No |
|  | Ex-smokers PI | OR = 2.18 (0.89 – 5.33) | No |
|  | Smokers PI | OR = 3.99 (2.58 – 6.16) | Yes |
|  | Non-smokers BI | OR = 1.19 (0.58 – 2.46) | No |
|  | Ex-smokers BI | OR = 0.37 (0.13 – 1.01) | No |
|  | Smokers BI | OR = 1.35 (0.57 – 3.24) | No |
|  | Non-smokers PD | OR = 0.3 (0.15 – 0.62) | Yes |
|  | Smokers PD | OR = 0.43 (0.14 – 1.31) | No |
| Zeng et al. (9) | COPD | OR = 2.08 (1.48 – 2.91) | Yes |
| Azarpazhooh et al. (10) | COPD | A weak association was identified between PD and COPD. | Yes |
| Scannapieco et al. (11) | COPD | Poor oral hygiene and PD are associated with respiratory diseases such as COPD. | Yes |
| Garcia et al. (12) | COPD | Poorer periodontal health increases the risk of developing COPD | Yes |

PD = Periodontal disease; COPD = Chronic obstructive pulmonary disease; OR = Odds ratio; MD = Mean difference; WMD = Weighted mean difference; ABL = Alveolar bone loss; OHI = Oral hygiene index; PD = Probing depth; PI = Plaque index; GI = Gingival index; RT = Remaining teeth; CLA = Clinical level and attachment; BI = Bleeding index; BP = Bleeding on probing

**References**

# Molina A, Huck O, Herrera D et al. The association between respiratory diseases and periodontitis: A systematic review and meta-analysis. *J Clin Periodontol* (2023) 50(6): 842-887. doi:10.1111/jcpe.13767.

# Yang M, Peng R, Li X et al. Association between chronic obstructive pulmonary disease and periodontal disease: a systematic review and meta-analysis. *BMJ Open* (2023) 13(6): e067432. doi:10.1136/bmjopen-2022-067432

# Wu Z, Xiao C, Chen F et al. Pulmonary disease and periodontal health: a meta-analysis. *Sleep Breath Schlaf Atm* (2022) 26(4): 1857-1868. doi:10.1007/s11325-022-02577-3.

# Kelly N, Winning L, Irwin C et al. Periodontal status and chronic obstructive pulmonary disease (COPD) exacerbations: a systematic review. *BMC Oral Health* (2021) 21(1): 425. doi:10.1186/s12903-021-01757-z

# Gomes-Filho IS, Cruz SS da, Trindade SC et al. Periodontitis and respiratory diseases: A systematic review with meta-analysis. *Oral Dis* (2020) 26(2): 439-446. doi:10.1111/odi.13228

# Mushtaq S, Ammaar M, Sajjad E. Association between respiratory diseases and oral health: A systematic review study. *Indo Am J Pharm Sci* (2019) 6(5): 10800-10807.

# Shi Q, Zhang B, Xing H et al. Patients with Chronic Obstructive Pulmonary Disease Suffer from Worse Periodontal Health-Evidence from a Meta-Analysis. *Front Physiol* (2018) 9:33. doi: 10.3389/fphys.2018.00033

# Tan L, Wang H, Pan C et al. Periodontal health and chronic obstructive pulmonary disease stratified by smoking: a meta-analysis. *Int J Clin Exp Med* (2016) 9(12): 23190-23197.

# Zeng XT, Tu ML, Liu DY et al. Periodontal disease and risk of chronic obstructive pulmonary disease: a meta-analysis of observational studies. *PloS One* (2012) 7(10): e46508. doi:10.1371/journal.pone.0046508

# Azarpazhooh A, Leake JL. Systematic review of the association between respiratory diseases and oral health. *J Periodontol* (2006) 77(9): 1465-1482. doi:10.1902/jop.2006.060010.

# Scannapieco FA, Bush RB, Paju S. Associations between periodontal disease and risk for nosocomial bacterial pneumonia and chronic obstructive pulmonary disease. A systematic review. *Ann Periodontol* (2003) 8(1): 54-69. doi:10.1902/anales.2003.8.1.54.

# Garcia RI, Nunn ME, Vokonas PS. Epidemiologic associations between periodontal disease and chronic obstructive pulmonary disease. *Ann Periodontol* (2001) 6(1): 71-77. doi:10.1902/anales.2001.6.1.71.
